# Histone Deacetylases in Neurodegenerative Diseases and Their Potential Role as Therapeutic Targets: Shedding Light on Astrocytes

**DOI:** 10.3390/ph18101471

**Published:** 2025-09-30

**Authors:** Pedro de Sena Murteira Pinheiro, Luan Pereira Diniz, Lucas S. Franco, Michele Siqueira, Flávia Carvalho Alcantara Gomes

**Affiliations:** 1 Laboratório de Avaliação e Síntese de Substâncias Bioativas, Instituto de Ciências Biomédicas, Universidade Federal do Rio de Janeiro, Rio de Janeiro 21941-902, RJ, Brazil; 2 Laboratório de Investigação Metabólica Associada ao Envelhecimento, Instituto de Ciências Biomédicas, Universidade Federal do Rio de Janeiro, Rio de Janeiro 21941-902, RJ, Brazil; luandinizrj@gmail.com; 3 Laboratório de Neurobiologia Celular, Instituto de Ciências Biomédicas, Universidade Federal do Rio de Janeiro, Rio de Janeiro 21941-902, RJ, Brazil

**Keywords:** astrocyte, aging, neurodegenerative diseases, HDACs, HDACi

## Abstract

Histone deacetylases (HDACs) are crucial enzymes involved in the regulation of gene expression through chromatin remodeling, impacting numerous cellular processes, including cell proliferation, differentiation, and survival. In recent years, HDACs have emerged as therapeutic targets for neurodegenerative diseases (NDDs), such as Alzheimer’s disease, Parkinson’s disease, and Huntington’s disease, given their role in modulating neuronal plasticity, neuroinflammation, and neuronal survival. HDAC inhibitors (HDACi) are small molecules that prevent the deacetylation of histones, thereby promoting a more relaxed chromatin structure and enhancing gene expression associated with neuroprotective pathways. Preclinical and clinical studies have demonstrated that HDACi can mitigate neurodegeneration, reduce neuroinflammatory markers, and improve cognitive and motor functions, positioning them as promising therapeutic agents for NDDs. Given the complexity and multifactorial nature of NDDs, therapeutic success will likely depend on multi-target drugs as well as new cellular and molecular therapeutic targets. Emerging evidence suggests that HDACi can modulate the function of astrocytes, a glial cell type critically involved in neuroinflammation, synaptic regulation, and the progression of neurodegenerative diseases. Consequently, HDACi targeting astrocytic pathways represent a novel approach in NDDs therapy. By modulating HDAC activity specifically in astrocytes, these inhibitors may attenuate pathological inflammation and promote a neuroprotective environment, offering a complementary strategy to neuron-focused treatments. This review aims to provide an overview of HDACs and HDACi in the context of neurodegeneration, emphasizing their molecular mechanisms, therapeutic potential, and limitations. Additionally, it explores the emerging role of astrocytes as targets for HDACi, proposing that this glial cell type could enhance the efficacy of HDACs-targeted therapies in NDD management.

## 1. Introduction

The global increase in life expectancy has led to a significant demographic shift, with a growing proportion of elderly individuals worldwide. As a result, the prevalence of age-related disorders, particularly neurodegenerative diseases (NDDs), has risen dramatically in recent decades. Among these, Alzheimer’s disease (AD) stands out as the most common and devastating form, representing a major public health concern.

AD is characterized by progressive cognitive decline, memory loss, and behavioral disturbances, and its impact extends beyond patients to include families, caregivers, and health systems. Despite considerable progress in understanding its molecular underpinnings, AD remains without a cure or truly effective disease-modifying treatment. This reflects, in large part, the complex and multifactorial nature of its pathophysiology, which involves a combination of genetic, epigenetic, biochemical, inflammatory, and metabolic factors.

Current pharmacological treatments, such as cholinesterase inhibitors and NMDA receptor antagonists, offer only limited symptomatic relief and do not alter the course of the disease [1]. This has led to an urgent need for new therapeutic strategies that go beyond symptomatic management and aim to interfere with the underlying mechanisms driving neurodegeneration. A growing body of evidence suggests that targeting a single molecular pathway may be insufficient to achieve meaningful clinical benefits in such complex disorders. In this regard, the development and investigation of multi-target compounds—capable of modulating several disease-relevant mechanisms simultaneously—has gained increasing attention [2]. These multi-functional drugs have the potential to address key features of AD pathogenesis, including synaptic dysfunction, protein aggregation (such as amyloid-beta and tau), oxidative stress, mitochondrial impairment, and chronic neuroinflammation. By acting on multiple fronts, these compounds may offer a more holistic and effective therapeutic approach.

Among the emerging targets for intervention, epigenetic regulation has stood out as a particularly promising field. Epigenetic mechanisms, including histone modifications and chromatin remodeling, control gene expression without altering the underlying DNA sequence. One of the key families of enzymes involved in this process is the histone deacetylases (HDACs), which remove acetyl groups from histone proteins, leading to a more condensed chromatin structure and reduced gene transcription. Their dysregulation has been associated with impaired synaptic plasticity, memory deficits, neuronal vulnerability, and neuroinflammation, processes that are central to AD and related disorders. Importantly, HDACs research does not stand in isolation but rather integrates into a broader framework of epigenetic-based therapeutic strategies, which aim to restore balanced gene expression patterns through multi-layered interventions. Consequently, histone deacetylases inhibitors (HDACi)—which promote chromatin relaxation and enhance the expression of neuroprotective genes—have emerged as promising candidates within this wider epigenetic therapeutic landscape for NDDs [3,4].

Preclinical studies using HDACi have demonstrated encouraging results, showing that these compounds can mitigate neuronal damage, reduce neuroinflammation, and improve cognitive performance in animal models of AD and other NDDs [3]. Some HDACi have already entered clinical trials, reflecting their therapeutic promise. However, while most of the early research has focused on neurons as the primary cellular target, more recent findings have highlighted the essential role of glial cells—particularly astrocytes—in the onset and progression of AD.

Astrocytes, long considered mere supportive cells in the central nervous system, are now recognized as highly dynamic and functionally diverse players in brain physiology and pathology. Beyond their trophic support to neurons, astrocytes engage in extensive physical and molecular crosstalk with other glial cells and the brain vasculature, exerting multifaceted roles in regulating brain function [5,6,7]. For instance, astrocytes contribute to synapse formation, pruning and transmission, regulate neurotransmitter uptake and recycling, maintain extracellular ion homeostasis and blood–brain barrier integrity, and actively participate in immune responses. In NDDs, astrocytes undergo profound morphological and functional changes, giving rise to heterogeneous reactive phenotypes that can exacerbate inflammation and disrupt neuronal communication [8,9]. Dysregulated astrocytic activity has been implicated in both early and late stages of the disease, reinforcing their relevance as potential therapeutic targets.

Importantly, HDACi have shown the ability to modulate astrocytic function, reducing their pro-inflammatory profile and restoring their neuroprotective capabilities [10,11]. By influencing astrocyte–neuron interactions, HDACi may promote a more favorable microenvironment for neuronal survival and function. This dual action—on both neurons and glia—positions HDACi as ideal candidates for multi-target interventions in multifactorial diseases like AD.

This review aims to provide a comprehensive overview of the therapeutic potential of HDACi in neurodegenerative diseases, with a particular emphasis on their effects in astrocytes and their implications for AD. Furthermore, it underscores the importance of advancing multi-target strategies that reflect the complex interplay of molecular and cellular mechanisms involved in disease progression. By expanding our therapeutic scope to include glial targets and embracing the development of multi-functional compounds, we may significantly enhance the efficacy and durability of treatments for Alzheimer’s disease and related neurodegenerative conditions.

## 2. HDACs Superfamily: Structural and Catalytic Framework

HDACs are classified into four groups based on their homology to yeast deacetylases, subcellular localization, and cofactor requirements: class I (HDACs 1, 2, 3, and 8), class IIa (HDACs 4, 5, 7, and 9), class IIb (HDACs 6 and 10), class III (sirtuins 1–7), and class IV (HDAC11) [12,13]. Among these, HDACs in classes I, II, and IV are zinc-dependent metalloenzymes, requiring Zn^2+^ in their active sites for deacetylation activity, while class III HDACs, known as sirtuins, require nicotinamide adenine dinucleotide (NAD^+^) as a cofactor, linking their activity to cellular energy status and metabolism [13,14,15]. A concise overview of HDACs classes, including their cofactors, subcellular localization, and representative functions, is provided in Table 1.

Class I HDACs are predominantly located in the nucleus, where they play a pivotal role in gene expression regulation, often being recruited to transcriptional repressor complexes [16,17,18,19]. For instance, HDAC1 and HDAC2 are commonly associated with co-repressors such as CoREST, NuRD, Sin3 and SMRT/NCoR, regulating the transcription of genes involved in cell proliferation, differentiation, apoptosis and beyond [20,21]. In contrast, class IIa HDACs (HDACs 4, 5, 7, and 9) are able to shuttle between the nucleus and the cytoplasm, a property regulated by phosphorylation-dependent interactions with 14-3-3 proteins [22,23,24]. Class IIa HDACs exhibit lower deacetylase activity compared to class I HDACs and are believed to act as scaffold proteins, integrating signals from different signaling pathways [22,23,24].

Regarding class IIb HDACs, histone deacetylase 6 (HDAC6) is a unique member of the HDACs family with distinct structural features. Unlike other HDACs, it contains two homologous catalytic domains (CD1 and CD2), both capable of deacetylation, although CD2 is more catalytically active [25]. This dual-domain arrangement is crucial for HDAC6 ability to target various non-histone proteins, such as α-tubulin and heat shock protein 90 (Hsp90) [26]. Another key feature is its ubiquitin-binding zinc finger domain, which allows HDAC6 to interact with ubiquitinated proteins, playing a role in protein degradation via aggresomes and autophagy [27,28,29]. HDAC6 is predominantly cytoplasmic, a characteristic facilitated by a nuclear export signal (NES) in its structure, enabling it to regulate cellular processes beyond transcription, such as cell motility and stress responses. This makes HDAC6 particularly important in processes like protein trafficking, cell motility, and protein degradation. It has also shown its relationship with NLRP3-inflammasome, in which HDAC6 selective inhibition attenuated LPS-induced IL-1β release both in vitro and in vivo by blocking the activation of NLRP3 [30]. Due to its role in these pathways, HDAC6 has emerged as a therapeutic target for diseases involving protein misfolding, including neurodegenerative disorders [31,32,33,34].

## 3. Pharmacophoric Features of HDACi

**Figure 1:**
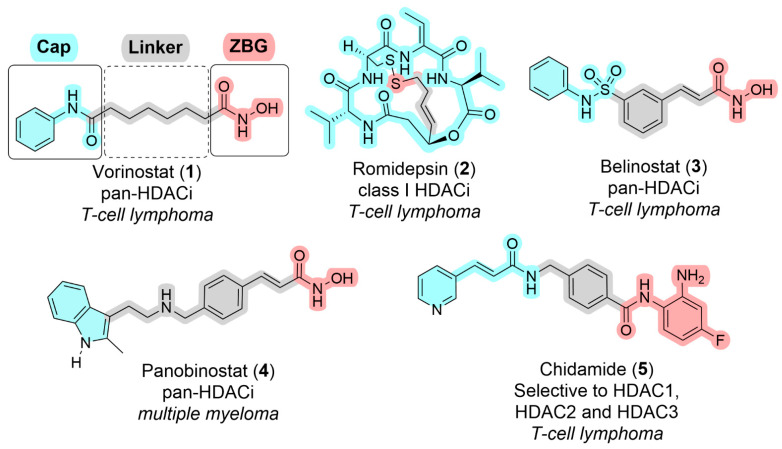
Pharmacophoric features and chemical structures of approved HDACi, highlighting their molecular targets and associated clinical indications.

Epigenetic modulation through HDACs inhibition has been intensively explored in oncology. HDACi lead to hyperacetylation of histones and non-histone proteins, resulting in various anti-cancer effects, including cell cycle arrest, differentiation, apoptosis, and inhibition of angiogenesis [35,36,37,38]. To date, four HDACi—vorinostat (**1**), romidepsin (**2**), belinostat (**3**), and panobinostat (**4**)—have been approved by the U.S. Food and Drug Administration (FDA) for treating hematologic malignancies, such as cutaneous T-cell lymphoma and multiple myeloma [39,40,41] (Figure 1). These drugs primarily target class I and class II HDACs, exerting their effects by altering gene expression, promoting tumor suppressor activation, and reducing pro-survival signaling in cancer cells. More recently, chidamide (**5**) (selective to HDAC1, HDAC2 and HDAC3) was approved by the China Food and Drug Administration (CFDA) for the treatment of peripheral T-cell lymphoma and is also in phase III clinical trials for the treatment of breast cancer (NCT02482753) and HIV infection (NCT02902185) (Figure 1).

HDACs inhibition is typically achieved through a well-characterized pharmacophore, composed of three key components: a zinc-binding group (ZBG), a linker, and a cap group. The ZBG interacts with Zn(II) in the active site of HDACs, while the linker occupies the HDACs channels and can be linear, cyclic, saturated, or unsaturated [42]. The cap group, usually hydrophobic and often aromatic, interacts with the enzyme’s protein surface, stabilizing the inhibitor [42].

Selectivity in HDACs inhibition is influenced by the structural differences among HDACs isoforms, particularly in their catalytic binding sites. While the core catalytic site of HDACs is highly conserved, the dimensions of the catalytic channels vary between different HDAC classes. For instance, the class I HDACs channel is deeper and narrower than that of class IIb HDACs, providing opportunities for selective targeting.

**Figure 2:**
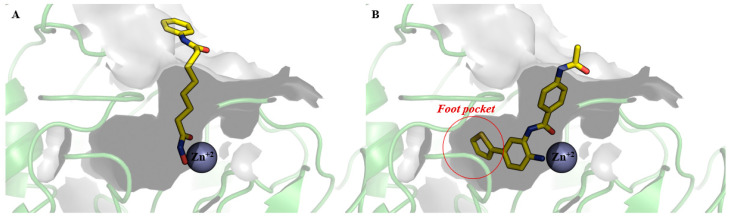
Comparative analysis of histone deacetylase 2 (HDAC2) bound to (**A**) the pan-HDAC inhibitor vorinostat (**1**) (PDB ID: 4LXZ) and (**B**) an *o*-aminoanilide derivative (PDB ID: 4LY1). In (**B**), the selective HDAC1/HDAC2 inhibitor, an *o*-aminoanilide derivative, engages additional interactions within the “foot pocket” of HDAC2, a structural feature specific to these isoforms, enhancing its selectivity. Small molecule carbons are shown in yellow.

One of the most used ZBGs in HDACi is the hydroxamic acid group, which strongly coordinates with the zinc ion in the active site. However, studies have shown that replacing hydroxamic acid with an *o*-aminoanilide group can confer selectivity for HDACs 1, 2, and 3 [43,44], resulting in the discovery of isoform-selective small molecules such as chidamide (**5**) (Figure 1). Further modifications of this ZBG, achieved by introducing heteroaromatic rings at position 4 of the *o*-aminoanilide scaffold, could allow engagement with an internal subcavity known as the foot pocket (Figure 2) within HDAC1 and HDAC2, thereby promoting selective inhibition of these isoforms over HDAC3 [45,46]. Figure 2 shows the comparative binding of vorinostat (**1**) (Figure 2A) and an *o*-aminoanilide derivative exploring additional interactions with the foot pocket of HDAC2 (Figure 2B) [46].

Binding kinetics also play a crucial role in the selectivity and efficacy of HDACi. *O*-aminoanilide-based inhibitors have been shown to exhibit a significantly longer residence time in the active site compared to hydroxamic acid-based inhibitors, which can enhance their therapeutic effects and reduce off-target interactions [46].

**Figure 3:**
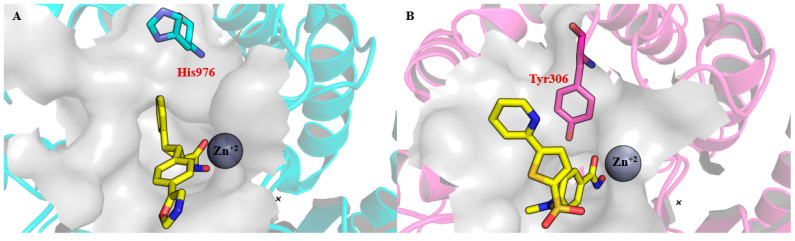
Selectivity pocket in HDAC4 in comparison to HDAC8. (**A**) A selective HDAC4 inhibitor bound to HDAC4 (PDB ID: 4CBY) interacts with the selectivity pocket unique to class IIa HDACs, formed by the substitution of a catalytic tyrosine with a histidine residue. (**B**) A hydroxamic acid derivative bound to HDAC8 (PDB ID: 1W22) highlights the influence of the catalytic tyrosine residue on the protein surface. Small molecule carbons are shown in yellow.

Selective inhibition of class IIa HDACs (HDACs 4, 5, 7, and 9) is also achievable through the exploitation of a distinct structural feature: a selectivity sub-pocket adjacent to the zinc-binding channel, unique to class IIa isoforms [47,48]. This sub-pocket is created by the substitution of the commonly conserved catalytic tyrosine residue in the active site of zinc-dependent HDACs for a histidine residue [24]. This structural modification distinguishes class IIa HDACs from other classes and is responsible for their lower deacetylase activity [22,23,24]. For example, structural comparisons between HDAC4 (PDB ID: 4CBY) [39] and HDAC8 (PDB ID: 1W22) [49] highlight this difference, with Tyr306 in HDAC8 replaced by His976 in HDAC4, allowing for isoform-specific targeting (Figure 3).

HDAC6 has been another focal point for selective inhibitor development due to its predominantly cytoplasmic localization and role in deacetylating non-histone proteins, such as α-tubulin and heat shock protein 90 (HSP90) [50,51,52]. HDAC6 inhibitors are particularly promising in the context of neurodegenerative diseases, given the enzyme’s lower toxicity profile compared to class I HDACs. Several structural features have been identified as key to HDAC6 selectivity: (i) the use of a phenyl linker [14,53,54], which fits into the unique HDAC6 channel; (ii) hydrogen bonding interactions with Ser531 at the channel entrance [55]; and (iii) the employment of bulky cap groups [14,56]. These strategies allow for selective inhibition of HDAC6 with minimal cross-reactivity with other isoforms, making it a safer option for long-term therapies.

Among the most used ZBGs in the design of HDAC6 inhibitors, hydroxamic acid is undoubtedly the most explored so far. However, issues with metabolic stability and the potential of hydroxamic acids to generate mutagenic and genotoxic metabolites—either through Lossen rearrangement or the release of hydroxylamine [57]—have driven the urgent need to identify safer ZBGs that can also provide selectivity for HDAC6.

**Figure 4:**
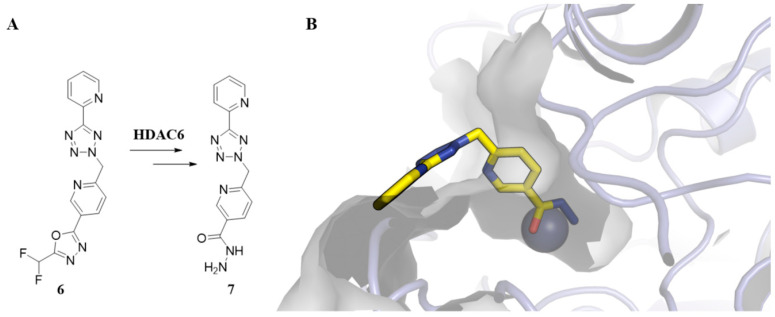
Binding mode of DFMO derivatives. (**A**) Formation of hydrazide (**7**) from DFMO (**6**) within the active site of HDAC6. (**B**) Binding mode of hydrazide (**7**) (carbons are shown in yellow) in HDAC6 (PDB: 8CJ7).

Among these new ZBGs, it is worth mentioning the 2-(difluoromethyl)-1,3,4-oxadiazole (DFMO) group, which has emerged as a promising alternative to hydroxamic acid in the design and development of HDAC6 inhibitors. It is also associated with good permeability and metabolic stability profiles [58]. DFMO-based HDAC6 inhibitors display nanomolar potency in the single-digit range, with an unprecedented selectivity exceeding 10^4^-fold for HDAC6 [59]. DFMO derivatives show a two-step slow-binding mechanism through a nucleophilic attack of the catalytic water molecule in the sp2 carbon of the DFMO group, resulting in a zinc-bound nitranion followed by deacetylation to form a hydrazide-bound derivative [60,61]. For example, Figure 4 shows the binding mode of the hydrazide derivative **7** (PDB: 8CJ7), formed by degradation of the corresponding DFMO derivative (**6**) in the active site of HDAC6.

Interestingly, while HDAC6 inhibitors have shown limited efficacy in cancer treatments, where cytotoxicity requires inhibition of multiple HDACs isoforms at higher concentrations, their potential in neurodegenerative disorders remains strong [62]. This is due to HDAC6’s cytoplasmic activity, primarily targeting non-histone substrates that are implicated in processes such as axonal transport and protein degradation [26,50,63]. The relatively lower toxicity of HDAC6 inhibitors further supports their use in chronic conditions like AD and PD [64].

By utilizing a combination of selective ZBGs, linkers and cap groups, and exploiting unique structural features of specific HDACs isoforms, the design of HDACi can be tailored for selective targeting, offering therapeutic benefits across a range of diseases with reduced off-target effects.

## 4. Implications of HDACs/HDACi in NDDs

**Figure 5:**
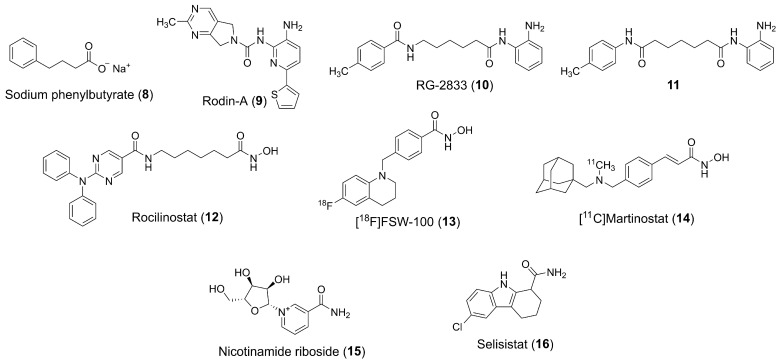
Chemical structure of HDACi under development for the treatment of neurodegenerative diseases.

Various agents, such as the hydroxamic acid-based compound vorinostat (**1**), have been investigated in the context of NDDs, including AD (phase I, NCT03056495) and Niemann-Pick disease (phase I/II, NCT02124083) [65]. Phenylbutyrate (PBA) (**8**) (Figure 5), a carboxylic acid derivative with modest HDACs inhibitory activity, has also been studied in combination with tauroursodeoxycholic acid (AMX0035), showing encouraging results for the treatment of amyotrophic lateral sclerosis, with FDA approval in 2022 after a successful phase II trial in the U.S. (NCT03127514) [66] and reaching phase III clinical trials (NCT05619783) in Europe. This fixed combination, AMX0035, has also been studied in Phase II trials for AD-related cognitive decline (NCT03533257) and HD (NCT00212316) [65].

Several pharmaceutical companies are actively recruiting for trials with other drug candidates targeting HDACs. One area of interest is the class I HDACs, which form part of multiprotein co-repressor complexes, including CoREST, NuRD, Sin3, and SMRT/NCoR, through which they modulate chromatin structure and deacetylate histone and non-histone proteins [67].

Notably, the CoREST complex is preferentially associated with HDAC2 in the brain, playing a pivotal role in repressing neuronal gene expression. Targeting HDACs within the CoREST complex is hypothesized to reduce peripheral toxicity while preserving neuroprotective effects. For instance, selectively inhibiting the CoREST complex with Rodin-A (**9**) (Figure 5) was shown to enhance synaptic density, increase the expression of synaptic proteins, and improve long-term potentiation in a mouse model, with doses that support long-term treatment safety [43]. In this context, Rodin Therapeutics (now Alkermes) has developed RDN-929 (structure not disclosed), an orally bioavailable HDAC-CoREST selective inhibitor [68,69]. The compound has undergone early clinical trials in healthy individuals, with the goal of developing it for the treatment of synaptopathies such as AD and PD (NCT03963973). Another compound, ALKS-1140 (structure not disclosed), a selective HDACi targeting the CoREST complex, is also in early clinical development (NCT05019105) by Alkermes as an oral therapy for neurological conditions. MBF-015 (structure not disclosed), a small molecule HDAC1/2 inhibitor under development by Medibiofarma, was tested for HD in adult patients and is in phase II trials (NCT06469853) [70].

For HD, the pimelic diphenylamide analogue RG-2833 (**10**) (Figure 5) has shown efficacy in mouse models [71] and has been investigated using the Patch-seq system to assess its effects on inhibitory GABAergic synaptic transmission and transcription at the single-neuron level [72]. Additionally, RG-2833 (**10**) has demonstrated effectiveness in animal models of Parkinson’s disease [73].

HDACi have also been studied for Friedreich’s ataxia, a neurodegenerative disorder caused by a significant reduction in frataxin levels, due to a large GAA triplet repeat expansion within the first intron of the frataxin gene (FXN). HDACi of the 2-amino-benzamide class, such as pimelic diphenylamide derivative (**11**), which selectively target HDAC3, have been shown to decondense the chromatin at the FXN locus and restore frataxin levels in Friendeich’s ataxia (FRDA) patient-derived cells and in mouse models of the disease, offering a promising approach for treating Friedreich’s ataxia [65,74,75]. In this regard, RG-2833 (**10**) entered phase I clinical development at Repligen for the oral treatment of Friedreich’s ataxia. A study involving FRDA patients dosed across four cohorts (30–240 mg/day) demonstrated increased FXN mRNA levels and H3 lysine 9 acetylation in peripheral blood mononuclear cells [76].

Other HDACi have been investigated for different applications. CKD-510 (structure not disclosed), an HDAC6 inhibitor in early clinical development, was tested for Charcot-Marie-Tooth disease. Although the compound’s structure has not been fully disclosed, patents suggest it may belong to the DFMO class of HDACi. Two phase I trials have recently been completed for this candidate (NCT05526742, NCT04746287). Another compound, EVP-0334 (FRM-0334) (structure not disclosed), is an orally bioavailable, brain-penetrant HDACi in phase II trials, sponsored by FORUM Pharmaceuticals (formerly EnVivo), for the treatment of frontotemporal dementia with a granulin mutation (NCT02149160). Additionally, rocilinostat (**12**) (Figure 5), an orally bioavailable HDAC6 inhibitor, is in phase II trials at Regenacy Pharmaceuticals for the treatment of painful distal symmetric sensorimotor polyneuropathy in patients with type 1 or type 2 diabetes (NCT03176472) [17].

In the field of diagnostic tools, the nuclear imaging agent [^18^F]FSW-100 (**13**) (Figure 5), which targets HDAC6, was developed for the diagnosis of neurological disorders [77,78,79]. Likewise, [^11^C]martinostat (**14**) (Figure 5), a radiopharmaceutical for imaging histone deacetylases, is undergoing phase I trials for brain imaging (NCT03721211) [80].

Although sirtuin inhibitors are less extensively studied than traditional HDACi, they have also shown potential in treating neurodegenerative diseases. However, clinical efficacy remains to be demonstrated. For instance, nicotinamide riboside (**15**) (Figure 5) was tested in clinical trials for AD (NCT05617508), mild cognitive impairment (NCT03482167), and PD (NCT03816020) [81,82,83]. Additionally, selisistat (**16**) (Figure 5) has progressed to phase II trials for HD (NCT01521585), where it was found to be safe and well-tolerated in early-stage patients, although it did not affect circulating levels of soluble huntingtin [84]. Table 2 provides a summary of clinical trials evaluating various HDACi for the treatment of neurodegenerative diseases, including their respective compounds, targeted HDAC isoforms, and study phases.

A promising approach currently under investigation to enhance the efficacy of HDACi is the development of multitarget inhibitors [85,86]. Although these compounds are still in the early stages of development, several have shown notable enzymatic inhibition and demonstrated in vitro activity. In the case of AD, for example, Qin et al. introduced compounds that act as both HDACs and acetylcholinesterase inhibitors [80,85], and Lei Wang et al. reported dual inhibitors of HDAC6 and butyrylcholinesterase (BuChE) [87]. Additional multitarget compounds have been developed to target phosphodiesterases (PDEs) [88,89,90], glycogen synthase kinase 3 beta (GSK3β) [91], NMDA receptors [92], and, more recently, GPR40 receptors [93]. Furthermore, compounds that focus on mitigating oxidative stress have also been identified [94].

## 5. HDACs and Astrocytes

### 5.1. Astrocytes in the Health and Injured CNS

Astrocytes are remarkably resilient and heterogeneous glial cells that ensure brain homeostasis and neural function by orchestrating key physiological processes. These critical functions encompass metabolic and trophic support to neurons, ion and water balance, regulation of synapse formation and function, and blood–brain barrier (BBB) maintenance and integrity [9]. Astrocytes extensively branch to physically associate with neuronal synapse through perisynaptic astrocytic process (PAPs) and, on the opposite pole, anchor to brain blood vessels via highly specialized perivascular endfeet, covering approximately 99% of the vascular surface [95]. This physical association allows astrocytes to function as an interface coupling neuronal activity to vascular supply [96,97], and also to facilitate the removal of harmful neuronal metabolites to the bloodstream. In PAPs, astrocytes play a crucial role in preventing glutamate excitotoxicity by clearing excess synaptic glutamate and converting it into glutamine, which is then recycled back to neurons to sustain excitatory neurotransmission [98,99]. In addition, astrocytes are primary sources of several soluble modulators through which they regulate synapse formation, maturation, and plasticity, such as thrombospondins (TSPs) [100], hevin/SPARCL1 [101], TGF-β1 [7,102], glypican-4 and -6 [103], and cholesterol [104]. Astrocytes from distinct brain regions display differences in morphology, region-specific expression of synapse-modifying molecules, and functional properties in the healthy CNS, which are essential for maintaining neuronal homeostasis and may underlie the increased regional susceptibility observed in certain neurological conditions [105,106].

Emerging evidence suggests that astrocytes respond dynamically to changes in their microenvironment, such as injury, inflammation, or disease, by adopting context-dependent reactive states [8]. These reactive astrocytes may lose their homeostatic functions or acquire new properties that can either exacerbate neuroinflammation in neurodegenerative conditions or, conversely, contribute to neural protection and tissue resilience, depending on the context and stage of the response. Astrocyte reactivity is highly heterogeneous and differs across brain regions, disease contexts, and stages. In response to these varying conditions, astrocytes undergo morphological changes and adopt distinct transcriptomic profiles that alter their neurotrophic functions and potentially influence the course of neurological disorders. This wide range of reactive states has prompted the classification of multiple astrocyte subtypes over time.

For many years, astrocytes were classified dichotomously as A2 (“beneficial”) and A1 (“harmful”) phenotypes, based on their presumed anti- and pro-inflammatory functions [107,108]. A1 astrocytes were identified as a neurotoxic reactive phenotype induced by activated microglia-derived cytokines, including IL-1α, TNF-α, and C1q. These astrocytes lose most regular astrocytic functions, including their capacity to promote neuronal survival and synaptogenesis. Expression of complement component 3 (C3) was identified as a hallmark of A1-like astrocytes and has also been detected in the aging brain and in several neurodegenerative diseases, such as AD, PD, and HD [107,109,110]. Interestingly, endothelial cells (ECs) activated by pro-inflammatory stimuli, such as lipopolysaccharide (LPS), can release toxic mediators that modulate astrocytic gene expression and induce a neurotoxic reactive state resembling the A1 phenotype [111]. Although these astrocytes exhibit increased C3 expression, their molecular profile differs slightly from that of microglia-induced A1 astrocytes originally described in earlier studies. A notable distinction is the upregulation of extracellular matrix genes, such as decorin, which has been associated with increased BBB permeability [112]. These findings underscore that, depending on the nature of the insult and the cellular interactions in the neural niche, astrocytes can acquire distinct transcriptomic signatures, even when classified under the same A1 designation. Moreover, as extensively explored in recent studies, this reinforces the notion that the binary classification is insufficient to capture the broad spectrum of reactive states that astrocytes can undergo.

In this regard, another astrocyte subtype with a distinct transcriptomic signature that is molecularly distinct from A1-like astrocytes has been recently described in the context of aging and AD. These so-called autophagy-dysregulated astrocytes (APDAs) are characterized by the abnormal accumulation of autophagosomes within swollen processes, resulting from defective regulation of key proteostasis pathways, including mTOR signaling, proteasomal activity, and lysosomal function [113]. Therefore, these astrocytes show defective protein trafficking and secretion, including reduced release of synaptogenic molecules such as Hevin and SPARC, ultimately failing to properly regulate synapse formation and elimination in the aged hippocampus. Supporting this, Hevin expression consistently decreases in distinct astrocyte subpopulations of AD patients [114,115]. Interestingly, we have described that astrocyte-specific overexpression of Hevin mitigates cognitive decline and enhances pre and postsynaptic marker colocalization in both aging and AD models [115]. Notably, the emergence of APDAs is significantly accelerated in APP/PS1 mice compared to age-matched wild-type controls, suggesting that amyloid-β may act as an inducer of autophagy dysfunction and contribute to the establishment of a distinct astrocytic state in neurodegenerative conditions.

Additionally, Habib and colleagues identified a distinct astrocyte subpopulation in aged wild-type mice, aged human brains, and 5xFAD mice, an AD model, termed disease-associated astrocytes (DAAs) [116]. DAAs represent a unique reactive astrocyte phenotype localized near amyloid-β (Aβ) plaques in AD, which emerges prior to the onset of cognitive decline and increases in abundance as the disease progresses. These GFAP-high astrocytes exhibit a distinct molecular profile, marked by elevated expression of genes related to endocytosis, the complement cascade, aging, and impaired Aβ clearance. Moreover, the transition from homeostatic to disease-associated astrocytes is accompanied by a progressive increase in the gene that encodes apolipoprotein E (APOE), which under pathological conditions impairs Aβ clearance and contributes to toxic plaque accumulation [116,117].

Interestingly, increased Apoe gene expression was also a feature of a distinct subcluster of reactive astrocytes identified in patients with temporal lobe epilepsy (TLE), named lipid-accumulated reactive astrocytes (LARAs) [118]. LARAs accumulate lipid droplets transferred from overexcited neurons in an APOE-dependent manner. This lipid overload impairs glutamate uptake by astrocytes, thereby exacerbating neuronal hyperexcitability, worsening seizure severity in TLE mouse models, and thereby contributing to disease progression.

**Figure 6:**
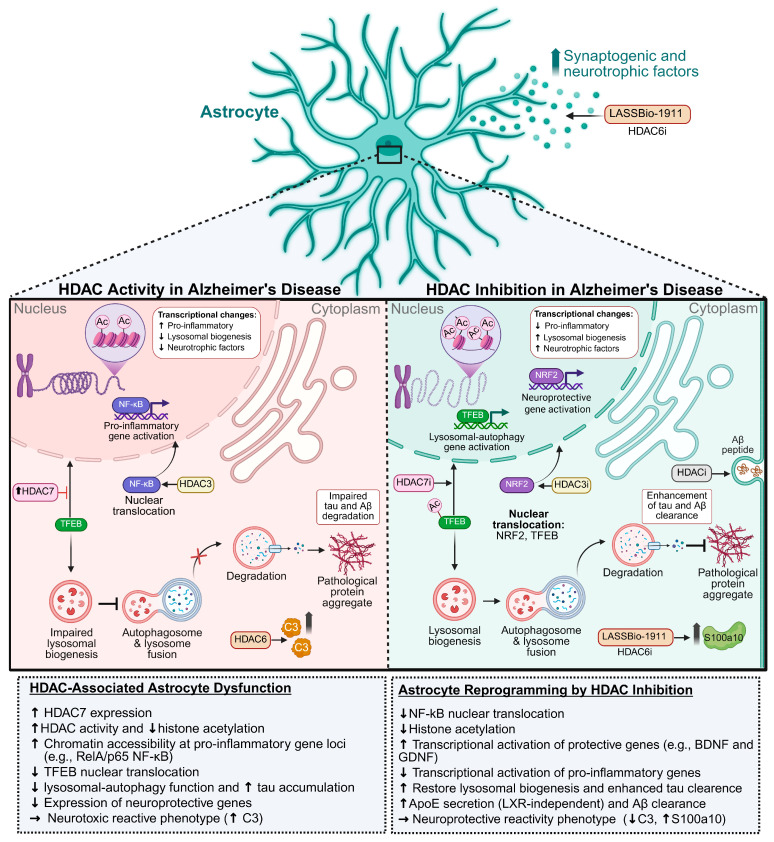
HDACs-driven epigenetic modulation of astrocyte phenotypes in AD. In AD, HDACs promote the transcriptional activation of pro-inflammatory genes through NF-κB nuclear localization and repress lysosomal biogenesis via TFEB deacetylation (**left panel**). These changes contribute to protein aggregation and drive a neurotoxic reactive state. HDACi shift astrocytes toward a neuroprotective phenotype by promoting the nuclear translocation of TFEB and NRF2, upregulating neuroprotective gene programs, restoring autophagy function and increasing Aβ clearance (**right panel**).

Astrocytes can adopt a wide range of transcriptomic and functional states in neurodegeneration, challenging the traditional binary view of reactivity as either neurotoxic or neuroprotective. A recent study shed light on this heterogeneity by identifying nine distinct astrocyte subpopulations (clusters 0–8) in human postmortem prefrontal cortex samples from AD cases, each defined by a unique transcriptional signature [114]. Some subtypes, such as clusters 0, 4, and 8, were enriched in genes involved in synaptic organization and transmission. Others, including clusters 2 and 5, expressed transcripts like ADAMTSL3, related to extracellular matrix remodeling and vascular integrity. Notably, certain astrocyte populations also showed elevated expression of genes associated with cell death, oxidative stress, and AD-risk genes, such as APOE and CLU. These findings highlight the coexistence of neuroprotective and neurotoxic astrocyte phenotypes within the same pathological environment and indicate that their relative prevalence may dynamically change across different disease stages and contexts. However, little is known about the molecular mechanisms underlying the transcriptional plasticity of astrocyte states. One relatively unexplored possibility involves the modulation of epigenetic machinery, particularly acetylation process. Among these, HDACs have emerged as key regulatory axes that orchestrate widespread transcriptional changes. Notably, modulation of HDACs activity can reprogram gene networks, thereby shaping distinct astrocyte phenotypes (Figure 6).

Cumulative evidence has shown that astrocytes switch from a healthy to a reactive state in response to proteotoxic stress induced by the accumulation of misfolded proteins, a pathological hallmark of several age-related NDs [119]. This dysfunction may be partially mediated by a decline in astrocytic autophagy–lysosomal efficiency, a critical degradation pathway for intracellular components, including protein aggregates. Disruption of autophagy in astrocytes enhance Aβ plaque formation and exacerbates key pathological features of AD in APP/PS1 mice [120]. In iPSC-derived astrocytes from PD patients carrying the LRRK2-G2019S mutation, impaired chaperone-mediated autophagy (CMA) and macroautophagy lead to progressive α-synuclein accumulation, ultimately contributing to non-cell-autonomous dopaminergic neurodegeneration [121]. However, the molecular mechanisms underlying astrocytic autophagy dysfunction in ND remain poorly understood.

Additional insight into this mechanism is provided by a recent study showing that astrocytic inhibition of HDAC7 improves lysosomal degradation and tau clearance via a transcription factor EB (TFEB) acetylation-dependent mechanism [122]. HDAC7 was found to be markedly upregulated in astrocytes from AD patients and PS19 transgenic mice, a tauopathy model. Specifically, TFEB deacetylation by astrocytic HDAC7 prevents its nuclear translocation, thereby impairing lysosomal biogenesis, which in turn compromises astrocyte-mediated tau degradation and worsens cognitive outcomes in mice. These findings uncover a previously unrecognized mechanism by which deacetylation may drive astrocytic autophagy dysfunction, contributing to the emergence of a disease-associated phenotype.

Another recent investigation uncovered that astrocyte reactivity triggered by IL-1α, TNF-α, and C1q induces widespread chromatin remodeling, increasing accessibility at genes involved in inflammatory pathways, including the RelA/p65 subunit of NF-κB [11]. Using a phenotypic chemical screening platform, HDAC3 was identified as a key epigenetic regulator of this cytokine-induced pathological reactive state. Selective HDAC3 inhibition in astrocytes suppressed the nuclear localization and pro-inflammatory transcriptional program of RelA/p65 NF-κB, while promoting the upregulation of neuroprotective astrocyte-associated genes downstream of nuclear factor erythroid 2-related factor 2 (NRF2). NRF2 signaling activation in astrocytes has been associated with neuroprotection in other neurodegenerative diseases [123,124].

Collectively, these findings highlight HDACs as potential therapeutic targets for modulating astrocyte states in neuroinflammatory conditions.

### 5.2. HDACs, HDACi and Astrocytes

HDACs exhibit distinct cell and region-specific expression patterns in the adult mouse brain, which are dynamically modulated by CNS injury. Class I HDACs (HDAC1–3) and class II HDACs (HDAC4 and HDAC6) are differentially expressed across neuronal and glial populations in cortical and hippocampal regions [125].

Among glial populations, astrocytes show specific expression patterns of HDACs isoforms. HDAC1 is found in both neurons and astrocytes, with astrocytic expression localized to the nucleus, cytoplasm, and end-feet, especially in the cingulate cortex, CA1, and subcortical white matter. HDAC2 is strongly nuclear in neurons and broadly distributed in astrocytes, including their end-feet, suggesting roles in structural and metabolic regulation. HDAC3, however, is more restricted in astrocytes, being absent in end-feet and undetected in regions such as the dentate gyrus and subcortical white matter, highlighting isoform-specific glial functions [125].

Beyond regional expression, HDACs are involved in regulating cell fate decisions. Valproic acid (VPA), a class I HDACi, promotes neuronal differentiation of adult hippocampal progenitor cells while suppressing astrocyte and oligodendrocyte differentiation—even under glia-favoring conditions. This effect is mediated by upregulation of neurogenic transcription factors such as NeuroD, suggesting that HDACs inhibition epigenetically shifts lineage commitment toward neurons [126].

HDAC3, in particular, plays a critical role in glial fate determination. Its deletion leads to increased astrocyte generation and reduced oligodendrocyte formation, indicating active suppression of the astrocytic program. HDAC3 cooperates with the co-activator p300 to promote oligodendrocyte-specific gene expression while repressing astrocytic regulators such as NFIA. Moreover, HDAC3 modulates STAT3 acetylation, further inhibiting astrocyte differentiation. These findings position HDAC3 as a key epigenetic regulator of glial identity [127].

Interestingly, while HDACs activity is required for oligodendrocyte maturation, it is dispensable for astrocyte development. In neonatal rat cortical progenitors, histone deacetylation occurs during oligodendrocyte differentiation, and treatment with trichostatin A (TSA) blocks this process. However, astrocyte differentiation remains unaffected, reinforcing the specificity of HDACs-dependent chromatin remodeling in oligodendrocytes [128].

Beyond development, HDAC3 also controls astrocyte reactivity in pathological contexts (Figure 6). It acts as a molecular switch for the inflammatory state of astrocytes. Inhibition of HDAC3 with RGFP966 reduces inflammatory gene expression and cytokine release, blocking the formation of pathological reactive astrocytes. In vivo, RGFP966 protects axons and reduces neuroinflammation in CNS injury models, highlighting HDAC3 as a therapeutic target for modulating astrocyte reactivity in neurodegenerative diseases [11].

In astrocytes, HDAC6 plays a central role in regulating inflammatory responses [129]. For example, in models of HIV-associated neuroinflammation, HIV-1 Tat protein induces HDAC6 expression, leading to reduced α-tubulin acetylation and upregulation of proinflammatory chemokines and adhesion molecules. HDAC6 knockdown or inhibition attenuates the activation of MAPK, NF-κB, and AP-1 signaling pathways, thereby dampening astrocyte-mediated inflammation [130]. Furthermore, HDAC6 is crucial for establishing inflammatory tolerance in astrocytes following repeated LPS stimulation. This effect is antagonized by GSK3, whose inhibition enhances HDAC6-dependent tolerance, indicating a functional balance between these pathways [131].

HDAC7 also contributes to astrocyte-mediated neuroinflammation. Upon LPS stimulation, HDAC7 interacts with IKKα/β to activate NF-κB signaling, thereby enhancing proinflammatory gene expression and inducing anxiety-like behaviors in animal models. Pharmacological or genetic inhibition of HDAC7 reverses these effects, underscoring its potential as a target in neuroinflammatory and neuropsychiatric disorders [132]. In AD, HDAC7 suppresses lysosomal biogenesis in astrocytes by deacetylating TFEB, a master regulator of lysosomal gene expression. This impairs tau clearance and contributes to astrocyte dysfunction. HDAC7 inhibition restores TFEB activity and improves cognitive performance in AD models [122].

Epigenetic modulation by HDACs also affects cytoskeletal and structural genes in astrocytes. For example, TSA and sodium butyrate alter GFAP splicing, increasing the GFAPδ/GFAPα ratio, which disrupts the intermediate filament network, mimicking features seen in Alexander disease [133].

Traumatic brain injury is another context where HDACs dysregulation contributes to pathology. In rat models of blast-induced neurotrauma, reduced acetylation of histones H2B, H3, and H4 in the prefrontal cortex correlates with persistent astrocyte activation and cognitive deficits. Notably, hypoacetylation is most prominent in astrocytes, suggesting their involvement in long-term neuroinflammatory responses [134].

HDACi have been shown to regulate inflammation in glial cells, although their effects are context-, dose- and isoform-dependent. Broad-spectrum inhibitors such as TSA and suberoylanilide hydroxamic acid (SAHA) can paradoxically enhance proinflammatory cytokines like TNF-α and IL-1β at low doses, while promoting anti-inflammatory cytokines such as IL-10 at higher concentrations. MS-275 (a class I HDACi) reduces TNF-α, whereas MC1568 (a class II HDACi) increases it. Transcriptomic analyses confirm that SAHA modulates pathways like JAK-STAT in a concentration-dependent manner. These results emphasize the need for precision in HDACi dosing and target selection to achieve therapeutic anti-inflammatory effects [135].

SAHA also reduces IFN-γ-induced inflammation in astrocytes by blocking STAT3 phosphorylation and suppressing the chemokine I-TAC, while preserving ICAM-1 expression. SAHA-treated astrocytes are less neurotoxic, highlighting its therapeutic potential in dampening astrocyte-mediated neurotoxicity [136].

HDACs also regulate lipid metabolism and amyloid clearance in astrocytes through effects on apolipoprotein E (apoE). Inhibition of class I HDACs increases apoE secretion from astrocytes via an LXR-independent mechanism. siRNA knockdown of HDACs isoforms confirms this regulatory pathway [137]. In ApoE4-expressing astrocytes, HDAC4 represses the sodium/hydrogen exchanger, NHE6, leading to endosomal acidification, retention of the LRP1 receptor, and impaired Aβ clearance. HDACi restore NHE6 expression and normalize endosomal pH, rescuing Aβ clearance and mitigating astrocytic dysfunction in AD [138].

HDACs inhibition also reshapes astrocyte chromatin to activate neuroprotective genes. Compounds like VPA, SB, TSA, MS-275, and apicidin enhance active histone marks (e.g., H3K4me2/3) and increase HSP70 expression in astrocytes. This occurs via recruitment of the acetyltransferase p300 and transcription factor NF-Y to the HSP70 promoter, positioning astrocytes as critical effectors of epigenetically driven neuroprotection [139].

In addition, HDAC2 and HDAC3 regulate the expression of fibroblast growth factor 21 (FGF21) in astrocytes. Inhibition of these isoforms increases FGF21 expression and elongates astrocytic processes. Knockdown of FGF21 shortens these processes, while overexpression restores them, indicating a novel epigenetic mechanism by which HDACs shape astrocyte morphology and potentially their neurotrophic function [140].

Inflammatory signaling in astrocytes is also epigenetically modulated via cyclooxygenase-1 (COX-1). TSA treatment induces COX-1 mRNA and protein expression through a TSA-responsive promoter element, accompanied by increased histone H4 acetylation and prostaglandin E2 production. These findings underscore the contribution of HDACs to astrocyte-mediated inflammatory pathways [141].

In the context of HIV neuropathology, HDACs are implicated in the epigenetic repression of antiviral gene expression and latency maintenance. HDAC6 is induced by HIV-1 Tat in astrocytes, promoting inflammation through α-tubulin deacetylation and activation of MAPK and NF-κB pathways [130]. Class I HDACs and SU(VAR)3-9 suppress HIV transcription in astrocytes, maintaining the viral reservoir within the CNS [142].

Dimethyl fumarate (DMF), a treatment for multiple sclerosis, modulates HDACs activity in astrocytes. Initially, DMF increases HDAC1, 2, and 4 mRNA levels, but chronic exposure reduces protein levels and activity, enhancing nuclear acetylation and activation of the Nrf2 pathway. This epigenetic shift reprograms astrocytes to adopt anti-inflammatory profiles [143].

In models of white matter ischemia, HDACi improve axonal recovery and preserve cellular integrity in optic nerves subjected to oxygen-glucose deprivation. This effect correlates with enhanced astrocytic glutamate transporter expression, reduced excitotoxicity, and preservation of ATP levels and mitochondrial integrity [144].

HDACi such as VPA, SB, and TSA enhance the astrocytic expression of neurotrophic factors like glial cell line-derived neurotrophic factor (GDNF) and brain-derived neurotrophic factor (BDNF), which are critical for the survival of dopaminergic neurons. In midbrain neuron-glia cultures, HDACi treatment increased H3 acetylation at the GDNF promoter, supporting an epigenetic mechanism for neurotrophic gene induction. These findings underscore the potential of HDACs inhibition to promote neuroprotection via astrocyte-mediated support in diseases such as Parkinson’s [145].

In primary human astrocytes, non-selective HDACi such as TSA and VPA also suppress antiviral and proinflammatory responses triggered by toll-like receptor (TLR) activation. Specifically, TLR3 and TLR4-induced cytokines and chemokines are downregulated by HDACi treatment, although the degree of suppression varies across inflammatory mediators. These data highlight how HDACs inhibition can dampen astrocyte-driven innate immune responses, relevant to conditions like viral encephalitis or chronic neuroinflammation [146].

In neurotoxicity models, TSA improves astrocytic glutamate handling under stress conditions. In MPP^+^-treated astrocyte cultures, TSA at concentrations ≥132 nM prevents the MPP^+^-induced elevation of extracellular glutamate and promotes glutamate uptake. This is linked to the preservation of glutamate transporter expression (e.g., GLT-1), normally downregulated by MPP^+^. These results suggest that HDACs inhibition maintains astrocyte homeostatic functions under toxic stress, contributing to neuroprotection [147].

Finally, activation of δ-opioid receptors with SNC-121 in optic nerve head astrocytes decreases HDAC1, 2, 3, and 6 expression, increases histone acetylation, and reduces TNF-α and GFAP expression. These results suggest that δ-opioid signaling can epigenetically modulate astrocyte reactivity via HDACs downregulation [148].

Our group has demonstrated that intracerebroventricular injection of amyloid-β oligomers (AβO) in mice leads to marked synaptic loss, increased HDACs activity, reduced histone acetylation in memory-related brain regions, and a shift of astrocytes toward a reactive, pro-inflammatory phenotype characterized by elevated levels of pro-inflammatory cytokines. Notably, the selective HDAC6/HDAC 8 inhibitor, LASSBio-1911, when administered alone, shows no cytotoxic effects on either astrocyte or neuronal cultures and significantly increases synaptic density both in vitro and in vivo. Importantly, astrocyte cultures treated with LASSBio-1911 exhibit enhanced synaptogenic potential, indicating that the compound not only protects neurons but also reprograms astrocytes to support synaptic integrity. Mechanistically, LASSBio-1911 reduces HDACs activity and promotes histone acetylation, thereby preventing both synaptic and memory deficits induced by AβO. Furthermore, treatment with LASSBio-1911 induces a shift in astrocyte phenotype toward a neuroprotective state marked by increased expression of S100a10 and decreased levels of inflammatory cytokines in AβO-injected animals. Together, these findings highlight LASSBio-1911 as a promising therapeutic candidate capable of modulating epigenetic pathways, reversing glial reactivity, and preserving cognitive function in the context of AD [10].

## 6. Concluding Remarks and Future Perspectives

HDACi have emerged as powerful epigenetic modulators in NDD therapy due to their multifaceted neuroprotective effects: they promote gene expression beneficial to neurons, dampen neuroinflammation, and enhance synaptic plasticity. Crucially, beyond neuronal targets, accumulating data show that HDACs inhibition exerts profound control over astrocyte function—restoring their metabolic support, attenuating reactive phenotypes, and improving clearance of pathogenic proteins [11,122,145,149]. These advances compel a shift from neuron-centric treatments to glia-inclusive strategies, placing astrocytes at the core of future drug design.

Since NDDs are mostly multifactorial, single-target HDACi may not sufficiently counteract the diverse pathological processes of protein misfolding, oxidative stress, inflammation, and impaired cellular clearance. This highlights the pressing need for multitarget-directed HDACi, designed to act on HDACs along with enzymes or receptors involved in neuroinflammation, proteostasis, or mitochondrial dysfunction [150]. Notably, dual HDAC6/SIRT2 inhibitors, and HDAC/PDE9 hybrid compounds, have demonstrated improved efficacy in reducing amyloid pathology and supporting neuronal survival in preclinical AD [151].

Despite their therapeutic promise, HDACi face important limitations. A key challenge is achieving isoform selectivity within the HDACs family, as broad inhibition may disrupt essential physiological processes. Hydroxamic acid-based inhibitors, the most commonly studied chemical class, present intrinsic toxicity due to the potential release of hydroxylamine, a cytotoxic metabolite. Moreover, ensuring sufficient brain penetration remains a critical hurdle given the restrictive nature of the blood–brain barrier. These considerations underscore the need for careful structural design, safety evaluation, and selection of alternative chemotypes. Collectively, these factors may underlie the limited success of many HDACi clinical trials in NDDs. While most studies do not explicitly report reasons for trial failure, we hypothesize that insufficient target engagement, variability in patient response, and pharmacokinetic limitations contribute substantially.

Advancing the next generation of HDACi will require integrating pharmacological innovation with precision medicine. Omics-based approaches—such as transcriptomics, epigenomics, and single-cell profiling—can reveal patient- and cell-type-specific HDACs signatures, guiding selective interventions and identifying predictive biomarkers. Equally important is the development of novel chemical scaffolds beyond hydroxamic acids, including benzamides, thiols, cyclic peptides, and other zinc-binding groups, to improve selectivity, safety, and pharmacokinetic profiles.

Looking ahead, rational development of multi-target-directed HDACi should focus on optimizing brain penetrance, isoform selectivity, and astrocyte bioavailability. Combining HDACs inhibition with complementary mechanisms—such as autophagy enhancement, antioxidant support, or anti-inflammatory signaling—could maximize therapeutic impact while minimizing toxicity. Additionally, identifying molecular descriptors of astrocyte states (e.g., reactive versus supportive) will guide selective targeting of glial subtypes.

In summary, future therapeutic paradigms must transcend neuron-exclusive interventions, moving toward multi-target, astrocyte-informed approaches. Integrating HDAC-based epigenetic modulation with broader pharmacological actions represents a promising frontier in the development of disease-modifying therapies for Alzheimer’s, Parkinson’s, Huntington’s, and related NDDs.

## Figures and Tables

**Table 1:** Structural and functional features of histone deacetylases (HDACs) classes.

Class	Members	Cofactor	Localization	Key Features/Functions
I	HDACs 1, 2, 3, 8	Zn^2+^	Nucleus	Strong deacetylase activity; transcriptional repression; complexes with CoREST, NuRD, Sin3
IIa	HDACs 4, 5, 7, 9	Zn^2+^	Nucleus ↔ Cytoplasm	Low catalytic activity; shuttle between compartments; scaffold proteins
IIb	HDACs 6, 10	Zn^2+^	Predominantly cytoplasmic	HDAC6: dual catalytic domains, ubiquitin-binding; regulates α-tubulin, Hsp90, protein degradation
III	Sirtuins 1–7	NAD^+^	Nucleus, cytoplasm, mitochondria	Metabolism-linked; roles in stress response, longevity, neuroprotection

**Table 2:** Summary of Clinical Trials Investigating HDACi in NDDs.

Study ID (NCT)	Compound/HDACi	Target/HDACs	Disease/Indication	Phase
NCT03056495	Vorinostat (**1**)	Pan-HDAC (hydroxamic acid)	AD	Phase I
NCT02124083	Vorinostat (**1**)	Pan-HDAC	Niemann-Pick disease	Phase I/II
NCT03127514	Phenylbutyrate (**8**) + Tauroursodeoxycholic acid (AMX0035)	Modest HDAC inhibition	ALS	Phase II
NCT05619783	Phenylbutyrate (**8**) + Tauroursodeoxycholic acid (AMX0035)	Modest HDAC inhibition	ALS	Phase III
NCT03533257	Phenylbutyrate (**8**)	Modest HDAC inhibition	AD	Phase II
NCT00212316	Phenylbutyrate (**8**)	Modest HDAC inhibition	HD	Phase II
NCT03963973	RDN-929	HDAC-CoREST selective	AD, PD	Early clinical trials
NCT05019105	ALKS-1140	HDAC-CoREST selective	-	Phase I
NCT06469853	MBF-015	HDAC1/2	HD	Phase II
-	RG-2833 (**10**)	HDAC1/3	Friedreich’s ataxia	Phase I
NCT05526742	CKD-510	HDAC6	Charcot-Marie-Tooth disease	Phase I
NCT04746287	CKD-510	HDAC6	Charcot-Marie-Tooth disease	Phase I
NCT02149160	EVP-0334 (FRM-0334)	Brain-penetrant HDACi	Frontotemporal dementia (granulin mutation)	Phase II
NCT03176472	Rocilinostat (**12**)	HDAC6	Painful diabetic peripheral neuropathy	Phase II
NCT03721211	[11C]Martinostat (**14**)	HDACs	Brain imaging	Phase I
NCT05617508	Nicotinamide riboside (**15**)	Sirtuin	AD	Phase I
NCT03482167	Nicotinamide riboside (**15**)	Sirtuin	Mild cognitive impairment	Phase I/II
NCT03816020	Nicotinamide riboside (**15**)	Sirtuin	PD	Phase I
NCT01521585	Selisistat (**16**)	Sirtuin	HD	Phase II

## Data Availability

The original contributions presented in this study are included in the article. Further inquiries can be directed to the corresponding authors.

## Abbreviations

The following abbreviations are used in this manuscript.

APDA	autophagy-dysregulated astrocytes
APOE	apolipoprotein E
Aβ	amyloid-beta peptides
BBB	blood–brain barrier
BDNF	brain-derived neurotrophic factor
C3	complement component 3
CD	catalytic domain
CNS	central nervous system
CFDA	China Food and Drug Administration
DAA	disease-associated astrocytes
DMF	dimethyl fumarate
FDA U.S.	Food and Drug Administration
FGF21	fibroblast growth factor 21
GCIs	glial cytoplasmic inclusions
FRDA	Friendeich’s ataxia
FXN	frataxin
GDNF	glial cell line-derived neurotrophic factor
GFAP	glial fibrillary acidic protein
GSK3β	glycogen synthase kinase 3 beta
HDACs	histone deacetylases
HDACi	histone deacetylase inhibitors
HATs	histone acetyltransferases
H4	histone 4
HD	Huntington’s disease
Hsp90	heat shock protein 90
LARA	lipid-accumulated reactive astrocyte
LBs	Lewy bodies
LPS	lipopolysaccharide
MPTP	1-methyl-4-phenyl-1,2,3,6-tetrahydropyridine
NAD^+^	nicotinamide adenine dinucleotide
NDDs	neurodegenerative diseases
NES	nuclear export signal
NRF2	nuclear factor erythroid 2-related factor 2
PD	Parkinson’s disease
PET	positron emission tomography
PBA	Phenylbutyrate
AD	Alzheimer’s disease
ALS	amyotrophic lateral sclerosis
PDEs	phosphodiesterases
SAHA	suberoylanilide hydroxamic acid
TFEB	transcription factor EB
TLE	temporal lobe epilepsy
TSA	trichostatin A
TSP	thrombospondins
VPA	valproic acid
ZBG	zinc-binding group
